# Efficiency and Usability of a Near Field Communication-Enabled Tablet for Medication Administration

**DOI:** 10.2196/mhealth.3215

**Published:** 2014-06-02

**Authors:** Adam Landman, Pamela M Neri, Alexandra Robertson, Dustin McEvoy, Michael Dinsmore, Micheal Sweet, Anne Bane, Sukhjit S Takhar, Stephen Miles

**Affiliations:** ^1^Brigham and Women's HospitalDepartment of Emergency MedicineBoston, MAUnited States; ^2^Harvard Medical SchoolBoston, MAUnited States; ^3^Information SystemsPartners HealthCareWellesley, MAUnited States; ^4^Brigham and Women's HospitalBoston, MAUnited States; ^5^Massachussetts Institute of TechnologyCambridge, MAUnited States

**Keywords:** medication systems, medication errors, mobile applications, automatic data processing, nursing

## Abstract

**Background:**

Barcode-based technology coupled with the electronic medication administration record (e-MAR) reduces medication errors and potential adverse drug events (ADEs). However, many current barcode-enabled medication administration (BCMA) systems are difficult to maneuver and often require multiple barcode scans. We developed a prototype, next generation near field communication-enabled medication administration (NFCMA) system using a tablet.

**Objective:**

We compared the efficiency and usability of the prototype NFCMA system with the traditional BCMA system.

**Methods:**

We used a mixed-methods design using a randomized observational cross-over study, a survey, and one-on-one interviews to compare the prototype NFCMA system with a traditional BCMA system. The study took place at an academic medical simulation center. Twenty nurses with BCMA experience participated in two simulated patient medication administration scenarios: one using the BCMA system, and the other using the prototype NFCMA system. We collected overall scenario completion time and number of medication scanning attempts per scenario, and compared those using paired *t* tests. We also collected participant feedback on the prototype NFCMA system using the modified International Business Machines (IBM) Post-Study System Usability Questionnaire (PSSUQ) and a semistructured interview. We performed descriptive statistics on participant characteristics and responses to the IBM PSSUQ. Interview data was analyzed using content analysis with a qualitative description approach to review and categorize feedback from participants.

**Results:**

Mean total time to complete the scenarios using the NFCMA and the BCMA systems was 202 seconds and 182 seconds, respectively (*P*=.09). Mean scan attempts with the NFCMA was 7.6 attempts compared with 6.5 attempts with the BCMA system (*P*=.12). In the usability survey, 95% (19/20) of participants agreed that the prototype NFCMA system was easy to use and easy to learn, with a pleasant interface. Participants expressed interest in using the NFCMA tablet in the hospital; suggestions focused on implementation issues, such as storage of the mobile devices and infection control methods.

**Conclusions:**

The NFCMA system had similar efficiency to the BCMA system in a simulated scenario. The prototype NFCMA system was well received by nurses and offers promise to improve nurse medication administration efficiency.

## Introduction

### Background

Medication errors continue to represent a source of patient harm and can lead to increased health care utilization and costs [1-4]. Studies estimate the rate of adverse drug events (ADEs) to be between 6.5 and 15 ADEs per 100 hospital admissions with 28% to 75% of ADEs being preventable [5,6]. Of all hospital ADEs, 34% take place during medication administration, and less than 2% are caught before administration is complete [7,8].

Barcode-enabled medication administration (BCMA) coupled with an electronic medication administration record (e-MAR) has been shown to reduce nontiming medication administration errors by 41%, potential adverse drug events by 51%, and eliminate transcription errors [9]. During BCMA in our hospital, nurses scan the medication barcode and the barcode on the patient’s wristband to verify they are administering the right medicine to the right patient, at the right dose, at the right time, and by the right route. Finally, the nurse scans their hospital identification badge to record the medication administration event in the medication administration record. While the sequence of scanning events may vary slightly between hospitals, all BCMA workflows require scanning the patient, the provider, and the individual medications.

### Importance

Medication administration is an essential clinical task for hospital-based nurses, accounting for up to 28% of nursing activity [10]. Using medications safely is a Joint Commission National Patient Safety Goal [11] and using assistive technologies in conjunction with e-MAR to track medications from order to administration is a requirement for hospitals to meet Stage 2 Meaningful Use of health care information technology [12]. Despite these important safety benefits and incentives for e-MAR use, there are reports of clinicians not using the barcode system or finding workarounds due, in part, to difficult to maneuver computers on wheels, barcode scanners that often require multiple scans, and devices that require clinicians to repeatedly identify themselves (login) and their patients [13]. Innovations in mobile technology may overcome some of these challenges and make e-MAR more efficient. Improving usability and saving just a few seconds with each medication administration could dramatically improve efficiency when extended to the millions of medication administration events that occur across all patients, providers, and hospitals.

### Goals of This Investigation

We developed a novel, next generation prototype near field communication-enabled medication administration (NFCMA) system taking advantage of a mobile device equipped with a reader for near field communication (NFC), a wireless communication protocol that allows secure exchange of small amounts of data by proximity or touch. We compared the efficiency and usability of this prototype NFCMA system with an established laptop BCMA system in high-fidelity simulated medication administration scenarios. With the ability to provide contactless identification of patient, provider, and medications and to enter data directly on the handheld mobile device, we hypothesized that NFCMA would be more efficient and usable than traditional BCMA solutions riding on “workstations on wheels” (WOWs).

## Methods

### Study Design and Setting

In this mixed-methods study, we evaluated the feasibility of a prototype NFCMA system, using a randomized cross-over study, a survey, and one-on-one interviews. We performed the study in an academic medical simulation center that provided a controlled environment without interruption, concern for patient privacy or the need to integrate our prototype with other clinical systems [14]. The Partners Health Care institutional review board (Partners Health Care, Boston, MA) approved this study.

Brigham and Women’s Hospital (BWH) is a leader in BCMA and custom developed a workstation-based BCMA that went into widespread hospital use in 2005. Licensed independent practitioners order medications through computerized provider order entry (CPOE). Nurses review electronic medication orders and retrieve ordered medications from the automated medication dispensing system. At the patient’s bedside, nurses use fixed workstations or WOWs with Bluetooth-linked handheld barcode scanners. The nurse scans barcodes on the medication (label affixed to medication packaging), the patient (hospital bracelet), and themselves (hospital identification badge). The system confirms the five rights of medication administration (right patient, right medication, right route, right time, and right dose) and alerts the nurse, as necessary, to prevent medication errors. The BCMA system then electronically records the medication administration event.

### Intervention: Pilot NFCMA System

We developed a prototype NFCMA system using the 7” Google Nexus. The Google Nexus was used because it was the first tablet available with the Android 4.0 (Ice Cream Sandwich) operating system version with support for NFC. Currently, there is a wide selection of commercially available mobile devices that include both NFC and application programming interfaces enabling custom application development. NFC standards for communications protocols and data exchange formats are based on existing radio-frequency identification (RFID) standards including ISO/IEC 14443 and FeliCa and the ISO/IEC 18092 modulation schemes [15]. When embedded in a mobile device, NFC technology allows a user to “tap” their mobile device to establish an electromagnetic data exchange with an NFC tag (which can be incorporated in a sticker) to collect information or register an action.

RFID technologies allow readers to communicate wirelessly with tags on objects to collect information about that object [16]. RFID operates across a spectrum of frequencies, each with different capabilities. In comparing different RFID data acquisition technologies it is important to note various frequencies and protocols that are options for automated information data collection using radio frequency. These can be divided into two categories, passive and active RFID. Active RFID tags have a transmitter that requires a power source (typically a battery), which are expensive to both charge and to replace. Passive tags draw power from electromagnetic waves transmitted by the reader that induce a current in the tag's antenna. Common frequencies used in passive RFID systems include [16]:

- Low-frequency: limited range and slow read rate (eg, tracking animals);
- High-frequency (HF): NFC, generally near-field electromagnetic data exchange in close proximity (eg, smartcard applications for secure identification and access control); and
- Ultra high frequency: 10-m high speed reader capability (eg, supply chain applications).

NFC HF passive RFID operates at the 13.56 MHz frequency, building on standards for smartcards including ISO/IEC 14443 and ISO 15693, which are already used in many hospitals to identify both providers and patients. NFC HF RFID works by close proximity of electromagnetic coupling (a couple millimeters), ensuring only one NFC tag is read at a time. For medication administration, we use a passive NFC tag that does not require an expensive power source (ie, a battery); the tag draws its power from electromagnetic waves transmitted by the reader that induce a current in the tag’s antenna. The close proximity (touch) required for NFC tag reading may be less difficult to achieve than the precise alignment, scan distances and issues with low light levels and/or print quality required for successful barcode reading. Further, the tap feature may resolve inefficiencies introduced by linear or two-dimensional barcodes that are difficult to scan when wrinkled or inadvertently torn. The availability of “off the shelf” NFC smartphones makes this technology an attractive and cost effective alternative to dedicated barcode reader/laptop workstations. For these reasons, NFC may be superior to bar codes and low frequency RFID for medication administration.

**Figure 1:**
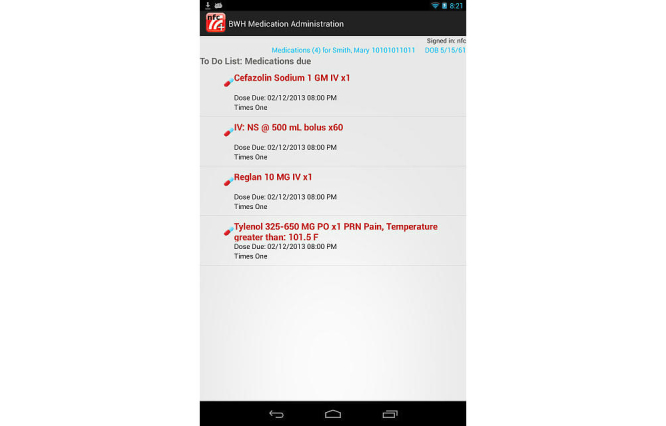
Prototype near field communication-enabled medication administration (NFCMA) system screenshot:medications due screen.

**Figure 2:**
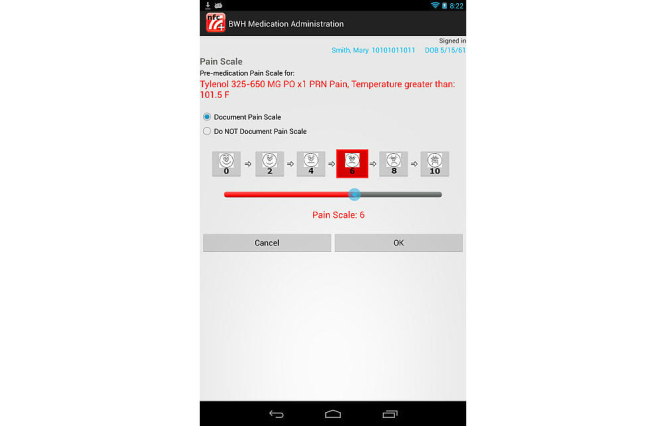
Prototype near field communication-enabled medication administration (NFCMA) system screenshot: pain scale dialog box.

The prototype NFCMA app was developed using the Android Software Development Kit. To ensure the study evaluated differences in mobile platform and NFC, the NFCMA software was designed to closely mimic existing barcode functionality; workflow and screens were similar to the current BCMA system. Differences were limited to the mobile footprint and the ability to identify the clinician, patient, and medication via NFC proximity touch. The mobile app also has the capability to confirm the five rights of medication administration and alert users with messages/warnings combined with sounds or vibration. Similar to the BCMA software, the NFCMA app prompted the user for additional information, including indication for “as needed” (PRN) orders, dose for variable dose medication orders, and pain level for pain medication orders. Importantly, the prototype NFCMA was developed as a standalone app; fictitious scenario data was hard coded into the app. The app did not capture real patient data or integrate with CPOE or other clinical information systems. Figures 1 and 2 show sample screenshots from the NFCMA app.

Instead of optical linear barcodes, NFC uses radio frequency tags encoded with unique identification numbers, such as the Global Trade Identification Number for medications. We used commercial off-the shelf NFC tags that were programmed and affixed to the hospital bracelet (patient identification [ID]), the hospital identification badge (nurse ID), and the medication packaging. We found medication blister packs interfered with NFC tag operation, so blister packs were placed in plastic bags and NFC tags were affixed to the plastic bags.

### Participant Selection

This pilot recruited 20 hospital-based nurses with BCMA experience. A sample size of 20 participants were selected given resource constraints and prior work establishing that 5 users are able to detect 85% of user interface issues [17,18] and 20 are able to detect 95% [19].

With the approval and assistance of nursing leadership, we identified email distribution lists including all hospital nurses. Given that this email distribution list included 2943 nurses, a 20.32% (598/2943) random sample of nurses was contacted with information about the study, including a frequently asked questions document explaining their role in the study and privacy/confidentiality statements. Participation was voluntary and had no effect on employment status or performance evaluations. Participants were selected based on order in which response was received and availability for study sessions. All participants provided verbal informed consent and were compensated US $100 for their participation in this study.

### Study Protocol

This study was conducted in a medical simulation center using a patient examination room that simulated a typical hospital room [14]. Each participant performed two simulated medication administration scenarios: one using the existing BWH BCMA system and the second using the prototype NFCMA system. Scenario order was randomized to minimize bias from carryover effects.

In collaboration with e-MAR process flow experts, we designed two simulated medication administration scenarios involving administration of a series of medications to a simulated hospitalized patient (Multimedia Appendix 1). To test the use of the e-MAR systems, the scenarios were designed to replicate typical inpatient medications and to include administration tasks similar to those typically performed with the existing BCMA system. Each scenario included a pain medication, an antiemetic medication, intravenous solution, and an antibiotic. A PRN order and variable dose range order were included in both scenarios, requiring the nurses to enter the dose administered and reason for administration on the barcode workstation or mobile device.

On arrival, study staff reviewed general study information with the participants and the participants provided verbal informed consent. Participants were instructed to complete medication administration as usual, identifying the patient, provider, and medication using the BCMA or NFCMA system. Prior to each scenario, nurses were provided a written script describing the clinical scenario and medication administration directives (Multimedia Appendix 1). Medications for each scenario were organized on a table in the simulated room. For the NFCMA scenario, nurses used the prototype NFCMA system and NFC tags were placed on each medication, patient ID bracelet, and nurse badge. For the BCMA control, nurses used standard hospital WOWs with handheld barcode scanner and the current version of our BCMA software using a test patient. Participants had the opportunity to review the script and ask questions before the scenario began.

Immediately before completing the NFCMA scenario, participants received a brief training on the NFCMA system using an additional scenario. This approximately 3-minute training session involved the administration of an antibiotic (Nitrofurantoin) and an analgesic (Phenazopyridine) to a patient with a urinary tract infection. Participants also practiced scanning the patient ID bracelet and nurse ID badges. Of note, Nitrofurantoin and Phenazopyridine were not included in the administration scenarios and NFCMA training did not include practice responding to allergy alerts, entering indications for PRN medications, or specifying the dose for medication orders with range dosing. Nurses did not receive training or practice with BCMA as the hospital e-MAR system currently in use, and on which these nurses have considerable experience, was used.

Study staff observed from a control room separated from the exam room by one-way glass. The patient in each scenario was a high fidelity mannequin with life-like functions including respiration, breath and bowel sounds, heart tones, pulse, and blood pressure. Nurses could talk to the patient mannequin and study staff would provide appropriate responses through the simulation center communication system.

After completion of both simulation scenarios, participants completed a Web-based survey covering demographic information, e-MAR experience, and the usability and workload of the prototype NFCMA system, through a modified International Business Machines (IBM) Post-Study System Usability Questionnaire (PSSUQ) [20]. Finally one research member (PN) conducted brief (10-15 minute), semistructured interviews with each participant, using an open-ended interview guide (Multimedia Appendix 2), to collect more detailed feedback on the experience and prototype NFCMA system, including scanning technology, portability/size, and workflow. The entire simulation encounter, including the one-on-one interview, was audio and video recorded using the simulation center’s audio-visual equipment and a backup digital audio recorder.

### Outcome Measures

Our primary outcome measures reflected efficiency and usability of the e-MAR systems. Efficiency was measured by the overall time to complete each medication administration scenario and the total number of scanning attempts per scenario. Overall time started when the participant entered the simulated patient’s room and ended when the nurse scanned her ID badge, the electronic signature and last step in the medication administration event. Total scanning attempts included scan attempts for patient ID, nurse ID, and medication. Under perfect circumstances, participants will have had seven scan attempts: one scan each for three medications; two scans for one medication that required two tablets; one scan for nurse hospital ID badge; and one scan for patient identification bracelet.

System usability refers to the efficiency, effectiveness, and satisfaction with which specific users can achieve a specific set of tasks in a particular environment [21]. We measured usability and workload of the prototype NFCMA system through a modified PSSUQ and from qualitative responses from the one-on-one interview.

### Data Collection and Analysis

Research study staff observed all participant sessions in real-time and recorded overall scenario time and scanning attempts. To standardize and to improve measurement, scanning attempt criteria were established (Table 1) and a trained observer (DM) reviewed video recordings of each session to confirm overall scenario completion time and total number of scanning attempts. Because several scenarios were interrupted by technical issues such as bugs in the tablet prototype, problems with previously entered medication orders in the barcode system, and other equipment issues, interruption start and end times were also recorded. Scenario times, interruption time, and scanning attempts were recorded in Microsoft Excel. Video recordings were not available for 2 participants, so these participant results were excluded from the time and scanning attempt results. For the analysis, we assumed these participants were missing completely at random.

Survey responses were collected using SurveyMonkey and transferred to Microsoft Excel for analysis. In addition to the video and audio recording, the interviewer and research assistants kept detailed notes of the one-on-one participant interviews, including key words and phrases used by participants.

Participant demographics were presented as descriptive statistics with frequency with percentage or mean for categorical and continuous data respectively. Participant’s overall time and total scanning attempts between the prototype NFCMA and BCMA systems were compared using a paired *t* test. We justified using parametric tests after viewing the differences graphically and using the Shapiro-Wilk test. We considered alternative distribution assumptions for the outcomes and analyzed the data using Wilcoxon signed-rank test and general estimating equations with bootstrap resampling. There were no contradictions between the *P* values from the paired *t* test compared with the nonparametric tests and general estimating equations; therefore, we report just the results of the paired *t* test for brevity and simplicity. In a sensitivity analysis, interruption time was subtracted from total time, to determine if interruptions impacted overall time. While interruptions did not impact number of scanning attempts, some participants entered their hospital identification number manually instead of scanning their barcode hospital ID badge. In order for hospital ID barcodes to be used with our barcode system, the badges must be activated. Some participants’ badges did not activate correctly and were not able to be scanned. Therefore, we performed an additional sensitivity analysis by adding a scan attempt to those participants who did not scan their hospital ID badge. Mean scores were summarized for each PSSUQ survey question. All statistical analysis was performed in Stata 12.0 and two-sided *P* values of less than 0.05 were considered to indicate statistical significance.

We performed a content analysis of the one-on-one interview data using a qualitative description approach, where notes from interviews and observations, supplemented with audio/video recordings, were reviewed, coded, and sorted to identify key phrases and meaningful text units as well as similarities and differences among the participants [22-24]. A subgroup of the research team (PN, AR, DM, and AL) met to discuss categories and subcategories of feedback, which were iteratively revised during the process. The group then selected representative quotes for each of the categories. Selected quotations were extracted from video recordings to ensure accuracy. Qualitative data was managed in Microsoft Excel.

**Table 1:** Scanning attempt criteria for barcode-enabled medication administration (BCMA) and near field communication-enabled medication administration (NFCMA) systems.

BCMA	NFCMA
Successful scan attempt	Successful scan attempt
Barcode scanner activation via button without a scan registering	Failed scan sound
Intentional barcode scanner manipulation	Intentional tablet manipulation (tapping or sliding) near NFC tag without a scan

## Results

### Participants

Twenty nurses with a mean of 14 years of nursing experience and 4.9 years of experience with the existing BCMA system participated in the study (Table 2). The majority (18/20, 90%) worked primarily on the medical/surgical floors and intensive care units, using the BCMA system during their shifts. Two participants worked primarily in the emergency department, but also serve as clinical educators and work with trainees on the medical/surgical units using and teaching BCMA. Almost all participants reported having their own computers (18/20, 90%) or smartphones (17/20, 85%), and 50% (10/20) reported owning tablets.

**Table 2:** Nursing participant characteristics (N=20).

Characteristic		n (%) or mean (range)
Nursing experience (years), range		14 (2-38)
Brigham and Women’s Hospital barcode-enabled medication administration (BCMA) experience (years), range^a^		4.9 (1-8)
**Nursing Unit Type, %**		
	Medical/surgical	15 (75)
	Intensive care unit	3 (15)
	Emergency department^b^	2 (10)
**Computer/Mobile Device Experience, %**		
	Own personal computer	18 (90)
	Own smartphone	17 (85)
	Own tablet	10 (50)

^a^One nurse excluded due to invalid data entry.

^b^These participants work primarily in the emergency department without BCMA, but also serve as clinical instructors on the medical surgical floors where they use (and teach) BCMA.

### Efficiency

Mean total scenario completion time was 202.4 seconds using the prototype NFCMA system compared with 182 seconds when using the BCMA system (Table 3). No statistically significant difference was observed (*P=*.09). Further, after adjusting for unplanned scenario interruptions, there was also no difference between total time using the prototype NFCMA system (188.2 seconds) and BCMA system (178.6, *P*=.32).

Mean scanning attempts were 7.6 using the prototype NFCMA system compared with 6.5 attempts using the BCMA system (Table 3). No statistically significant difference was observed (*P*=.12). In sensitivity analysis, the addition of one scan attempt to those using the barcode scanning system but manually entering their nurse ID yielded results that were even more similar to the mean NFC scan attempts (mean scan attempts 7.4, *P*=.80).

**Table 3:** Summary of quantitative results comparing the efficiency of the prototype near field communication-enabled medication administration (NFCMA) system to barcode-enabled medication administration (BCMA) system.

	NFCMA Mean (seconds) (SD)	BCMA Mean (seconds) (SD)	Mean difference	95% CI of the difference	*P* value
Total scenario completion time	202.4 (39.0)	182.0 (42.1)	20.4	−3.6-44.4	.09
Total scanning attempts	7.6	6.5	1.1	−.3-2.4	.12

### System Usability

Using a validated survey instrument, 19/20 (95%) participants agreed or strongly agreed that the prototype NFCMA system was easy to learn and use, enabled efficient task completion, and offered a pleasant interface (Table 4). Overall, 19/20 (95%) participants were satisfied with the prototype NFCMA system. No participants disagreed or strongly disagreed with any of the usability metrics.

Participants provided three major categories of feedback on the prototype NFCMA system during the one-on-one interviews: implementation and operational concerns, usability, and functional/feature enhancements. Table 5 summarizes representative quotes from nurse participants in these categories and subcategories.

Participants noted implementation concerns about how the prototype NFCMA system would be operationalized in the hospital environment. For example, participants expressed concern about no longer having the WOW, which they often use to transport medications to the patient. Participants also questioned how the tablets would be safely cleaned, disinfected, and stored.

In addition to implementation concerns, participants discussed issues relating to the usability of the tablet, including the current functionality, scanning, and interface of the tablet. While participants gave mixed feedback on the portability and size of the tablet as compared with the current system (Table 5), they were pleased with its ease of use. Participants provided constructive feedback about the sound and touch interface potentially being challenging in the clinical environment.

Finally, participants suggested enhancements to the prototype NFCMA system, such as a link to the hospital’s drug administration guide, which they have in their current BCMA system.

**Table 4:** Usability and workload of the prototype near field communication-enabled medication administration system, assessed through a modified International Business Machines Post Study System Usability Questionnaire.

Question	Strongly Agree ------------> Strongly Disagree							Rating average
	1	2	3	4	5	6	7	
Overall, I am satisfied with how easy it is to use this system.	13	6	0	1	0	0	0	1.45
It was simple to use this system.	14	5	1	0	0	0	0	1.35
I could effectively complete the tasks and scenarios using this system.	14	5	0	1	0	0	0	1.40
I was able to complete the tasks and scenarios quickly using this system.	12	7	0	1	0	0	0	1.50
I was able to efficiently complete the tasks and scenarios using this system.	12	7	0	1	0	0	0	1.50
I felt comfortable using this system.	12	7	1	0	0	0	0	1.45
It was easy to learn to use this system.	15	4	1	0	0	0	0	1.30
I believe I could become productive quickly using this system.	14	4	1	1	0	0	0	1.45
The organization of information on the system screens was clear.	15	4	1	0	0	0	0	1.30
The interface^a^ of this system was pleasant.	15	4	0	1	0	0	0	1.35
I liked using the interface^a^ of this system.	14	5	0	1	0	0	0	1.40
Overall, I am satisfied with this system.	13	5	1	1	0	0	0	1.50

^a^The “interface” includes those items that you use to interact with the system. For example, some components of the interface are the keyboard, the mouse, the microphone, and the screens (including their use of graphics and language).

**Table 5:** Summary feedback on the prototype near field communication-enabled medication administration (NFCMA) system by category with example participant quotes.

Category		Quotes
**Implementation concerns**		
	Transmission of infection	*This [tablet] could transmit bacteria from room to room.* [Participant 10] *I just wonder what would be done for infection control [when using the tablet].* [Participant 14]
	Cleaning tablet	*Are you going to be wiping it [the tablet] down every time you go to the patient?* [Participant 1] *The concern of if we are able to use the aseptic wipes to clean it [the tablet].* [Participant 17]
	Carrying meds and holding tablet	*I find that all the nurses will lay out all their meds on the cart [WOW* ^*a*^ *] and bring it into the patient’s room. And if you’re just holding this [tablet] I don’t know where everything else would go.* [Participant 20] *We’ve become attached to our computers in using the flat surface [of the WOW] to carry meds into the room, and maybe a cup of water or something for the patient.* [Participant 16]
	Dropping/losing/protecting the tablet	*Those [tablets] will get lost, they’ll get broken, they’ll get dropped.* [Participant 12] *Just a case or something to make it [the tablet] durable – because I can guarantee that it will get dropped.* [Participant 19]
	Tablet storage and availability	*I wonder when we are actually using them will we all have our own tablet.* [Participant 7] *I’m not sure if this [tablet] would be one device for all the patients*. [Participant 8]
**Usability**		
	Portability and size	*It [the tablet] seems kind of big though ... maybe it could be a little smaller.* [Participant 4] *It’s [the tablet] a nice size, nice and small.* [Participant 2] *I’m just afraid it’s [the tablet] a little too portable, in terms of just leaving it somewhere.* [Participant 8] *I like the fact that you don’t have to bring the computer into your room – you know it’s [the tablet] just a little more portable.* [Participant 1]
	Ease of use	*It [the tablet] was small, it was easy to use – it’s user friendly basically.* [Participant 12] *I think it [the tablet] was user-friendly obviously pending getting used to it, it’s just a matter of time.* [Participant 19]
	Tablet	*If it’s [the tablet] easy to touch with gloves on it would be fine – it would kind of be a pain to be taking on and off your gloves.* [Participant 3] *The beeps [from the tablet when scanning medications] are not loud though, when it does go through.* [Participant 5] *Some people would probably feel like that keyboard [on the tablet] is too small*. [Participant 14]
**Enhancements**		
	Access to additional applications/information	*Would we be able to get to the DAG [Drug Administration Guide] from here [tablet]? .... Often times some meds say it goes over three hours, but if you go on the DAG in an emergency case you can run it over an hour – it’s nice to have that immediately.* [Participant 11] *At least being able to get [on the tablet] the hospital nursing policies, and the pharmacy drug administration guidelines and MicroMedex would be really important for med administration.* [Participant 14]

^a^Workstations on wheels

## Discussion

### Principal Results

We developed a prototype e-MAR system using a tablet and NFC. In a pilot study conducted in a medical simulation center, we found no statistically significant difference in medication administration efficiency (total scenario time and scanning attempts) between the existing BCMA system and the prototype NFCMA system running on a mobile device. Nurse participants overwhelmingly found the prototype NFCMA system highly usable and offered next steps required for implementation. Given increasing attention to EHR efficiency and usability and the inclusion of e-MAR use as part of Stage 2 Meaningful Use, the mobile NFCMA platform or its components may eventually be an effective alternative to BCMA systems.

With only 3 minutes of training, participants were able to successfully complete all medication administration tasks using the prototype NFCMA system. Importantly, participants were able to complete these scenarios as efficiently with the prototype NFCMA systems they were with a BCMA system with which they had an average of 4.6-years’ experience. This is partly explained because the prototype NFCMA system was designed to have a similar look and feel to the existing BCMA system. Further, participants had considerable experience with mobile devices, which may have increased their ability to rapidly learn the prototype NFCMA system. These results further support the survey and qualitative findings that the prototype NFCMA system was well-designed and easy to use.

In addition to highlighting the high usability of the prototype NFCMA system, the qualitative interviews raised important barriers for use of this prototype NFCMA system as well as other mobile apps in the health care environment. There is increasing attention to reducing nosocomial transmission of infection, including those from inanimate objects, such as tablets. Previous work suggests that mobile devices can harbor infectious organisms [25,26]; however, safe ways of cleaning mobile devices have not been definitively described. Adding waterproof protective covers and building tablets using durable health care plastics allow tablets to be disinfected. Dedicating mobile devices to individual patient rooms is another possible solution to prevent spread of hospital-acquired infection. The infection risk with the prototype NFCMA system is similar to the current BCMA system. Finally, there must be attention to where tablets will be stored and how they will be charged. While seemingly minor details, if these implementation details are not defined in advance with attention to the workflow and efficiency implications, NFCMA and other mobile health care application implementations may have limited success.

### Comparison With Prior Work

While barcode technologies are well established in health care, this study is among the first assessing the potential for NFC apps to improve electronic medication administration. One early pilot assessed NFC as a tool for general nursing tasks and training, including e-MAR [27]. Other reports have used mobile phones with NFC to track self-reported patient outcomes [28] and medication compliance in both routine treatment and clinical trials [29]. Other work proposes more general health care apps for NFC [30]. These previous NFC health care app reports have limited evaluations, typically leverage mobile phones rather than tablet devices, and omit the efficiency and usability testing central to this evaluation.

### Limitations

This study has some important limitations. First, this was a prototype study conducted in a simulation setting. We attempted to replicate the clinical environment in a high-fidelity simulation center, but our results may not be generalizable to clinical settings. Second, this was a pilot study with a small sample size. We may not have had adequate power to detect small differences in time and scanning efforts between the two systems. Further, our scenarios were short, limiting our ability to detect differences that may be apparent with longer, more complicated clinical situations. Fourthly, participants experienced some technical difficulties during the encounters. While these unexpected events showed no impact on outcome measures in sensitivity analyses, they will need to be addressed prior to implementation. These technical difficulties were generally experienced with the prototype NFCMA system (coupled with brief training and experience with NFC) biasing the study toward the existing BCMA environment.

While NFC is a promising technology to improve medication administration, there are several limitations that must be overcome before this technology can be broadly applied to e-MAR. NFC supports ISO 14443 smartcard standards, therefore these devices can read patient and provider ID systems that may be deployed in hospitals today. What are missing are NFC labels for medications, which are currently labeled with optical barcodes. In our study, we manually labeled medications with NFC tags. This is time consuming and not feasible on a larger scale. Ideally, pharmaceutical manufacturers or distributors would incorporate NFC tags into product packaging, as is the current practice with barcodes. These NFC tags could be seamlessly incorporated into packing materials, and ideally identified with a common symbol. Further, the NFC tags could be printed on a label with a barcode and human readable medication identification number as a backup in case the NFC reader or tag failed.

Not all US mobile devices support NFC. However, NFC is estimated to have been included in one out of three smartphones sold in 2013, increasing total NFC-enabled devices to 400 million globally [31]. Broader support of NFC in mobile devices will increase availability and drive down costs. In the short-term, a subset of medications might be identified, such as specialty pharmaceuticals, where extra efforts to place NFC tags are outweighed by the benefits of product authentication and reliable recording of product/patient interactions.

### Conclusions

One important clinical tool that has been shown to reduce medication errors and potential adverse drug events at the point of medication administration is e-MAR [9]. Use of e-MAR is now a core measure of Stage 2 Meaningful Use, so e-MAR use will continue to increase over the next few years. NFCMA on a tablet device offers an alternative to traditional workstation-based BCMA. We found similar operational performance with improved usability in this prototype simulation study. While additional clinical studies and additional NFC/mobile operational tools will be required for future evaluations, NFCMA is a promising tool to improve medication administration.

## Multimedia Appendix 1

Clinical scenarios for prototype near field communication-enabled medication administration (NFCMA) system and barcode-enabled medication administration (BCMA) system.

## Multimedia Appendix 2

Qualitative interview guide.

## Abbreviations

ADE: adverse drug event
BCMA: barcode-enabled medication administration
BWH: Brigham and Women’s Hospital
CPOE: computerized provider order entry
e-MAR: electronic medication administration record
EHR: electronic health record NFC: near field communication
HF: high-frequency
IBM: International Business Machines
ID: identification
NFCMA: near field communication-enabled medication administration
PRN: “as needed”
PSSUQ: Post-Study System Usability Questionnaire
RFID: radio frequency identification
WOW: workstations on wheels
