# Supplementary material for: Efficiency and Usability of a Near Field Communication-Enabled Tablet for Medication Administration
Source: JMIR Mhealth Uhealth. 2014 Jun 2;2(2):e26. doi: 10.2196/mhealth.3215 (PMC4114445; doi:10.2196/mhealth.3215)
Supplement: Supplementary file 1 [file mhealth_v2i2e26_app1.pdf]

NFC Scenario

Post-op Total Knee Replacement with Cellulitis

Patient Name: xxxx, xxxxx

Patient ID: xxxxxxx

51 yo female with history of hypertension had a total knee replacement 2 days ago and developed a cellulitis over the surgical site. She remains in the hospital for IV antibiotics. She has an allergy to penicillin.

You are coming on to your shift and meeting the patient for the first time. She complains to you of a mild headache (similar to previous headaches) and nausea. You discuss with the on-call physician and jointly decide to give the patient the following medications now:

Note: Please give 650 mg Tylenol and administer all medications at one time. The patient tells you their pain level is at a 6.

To Do Orders (due now):

(IVF) NS 500 mL bolus IV x 60 minutes

(Abx) Cefazolin 1 gram IV x 1 now

Metoclopramide 10 mg IV x 1 now

Acetaminophen 325-650 mg PO q 8 hrs x 1 PRN pain/fever > 101.3

Barcode Scenario

Moderate Pancreatitis with Urinary Tract Infection

Patient Name: xxxx, xxxxx

ID: xxxxxxxx

55 yo female with history of coronary artery disease and of idiopathic pancreatitis admitted with acute pancreatitis and urinary tract infection. She remains in the hospital for pain control, GI tract rest, and IV antibiotics for her UTI. She has an allergy to penicillin.

You start your shift and evaluate the patient for the first time. She complains of nausea and a mild headache, but otherwise says she is starting to feel better. You discuss with the resident team. Please give the patient the following medications now:

Note: Please give Ibuprofen 400 mg for the headache and administer all medications at one time. The patient tells you their pain level is at a 4.

To Do Orders (due now):

(IVF) Lactated Ringers (LR) 1 L bolus IV x 60 minutes

(Abx) Ceftriaxone 1 gram IV x 1 now

Ondansetron 4mg IV x 1 now

Ibuprofen 200-800 mg PO q 8 hrs x 1 PRN pain/fever > 101.2
