# Supplementary material for: Efficiency and Usability of a Near Field Communication-Enabled Tablet for Medication Administration
Source: JMIR Mhealth Uhealth. 2014 Jun 2;2(2):e26. doi: 10.2196/mhealth.3215 (PMC4114445; doi:10.2196/mhealth.3215)
Supplement: Supplementary file 2 [file mhealth_v2i2e26_app2.pdf]

Qualitative Interview Guide

1. How was your experience using the BWH e-MAR system? What did you like? What did you dislike?
2. How did today's simulation compare with your usual e-MAR use in the hospital?
3. How was your experience using the mobile NFC e-MAR system?
   a. What did you like?
   b. What did you dislike?
4. Describe your experience scanning with NFC (medications, the patient and your badge)
   a. Is this the same or different than your experience with the barcode technology?
5. Describe your experience performing e-MAR using a tablet?
   a. How was the device size?
   b. How was the portability?
6. How does the mobile device fit into your medication administration workflow?
